# Real-world effectiveness and safety of carfilzomib, pomalidomide, and dexamethasone in relapsed/refractory multiple myeloma: a retrospective analysis from China

**DOI:** 10.3389/fonc.2026.1687341

**Published:** 2026-06-18

**Authors:** Wei Jiang, Wei Zhong, Jingdi Yu, Yufei Tang, Qunyi Guo

**Affiliations:** 1 Department of Hematology, Shangyu People’s Hospital of Shaoxing, Shaoxing, China; 2 Department of Hematology, Taizhou Hospital of Zhejiang Province Affiliated to Wenzhou Medical University, Linhai, China

**Keywords:** carfilzomib, dexamethasone, multiple myeloma, pomalidomide, real-world

## Abstract

**Background:**

Pomalidomide, a next-generation immunomodulatory drug (IMiD), and carfilzomib, a novel proteasome inhibitor (PI), have been separately approved in combination regimens for relapsed/refractory multiple myeloma (RRMM). However, real-world data, particularly for Chinese RRMM patients with prior-line treatment, including lenalidomide and bortezomib, remain limited.

**Methods:**

We retrospectively analyzed RRMM patients treated with KPd between October 2022 and December 2024 at two Chinese centers.

**Results:**

This study included 34 patients who received KPd. The median age was 65 years. All patients were bortezomib-exposed, 91.2% were lenalidomide-exposed (and refractory), and 50.0% were daratumumab-exposed. The overall response rate (ORR) was 82.4% and was significantly influenced by high-risk cytogenetics, prior daratumumab exposure, and the receipt of ≥ 3 prior lines of therapy. The median progression-free survival (PFS) was 13 months, with a 2-year PFS rate of 40.3%. Hematologic toxicities were the most common adverse events (AEs) (73.5%), and grade 3/4 AEs occurred in 58.8% of patients.

**Conclusions:**

KPd exhibited substantial effectiveness in this real-world cohort of heavily pretreated Chinese RRMM patients, including those with lenalidomide-refractory disease. However, the high incidence of hematologic toxicities necessitates careful monitoring and management.

## Introduction

Multiple myeloma (MM) is the second most common hematological malignancy worldwide (1, 2). The therapeutic landscape has been transformed by proteasome inhibitors (PIs), immunomodulatory drugs (IMiDs), anti-Cluster of Differentiation 38 (CD38) monoclonal antibodies (e.g., daratumumab and isatuximab), and emerging cellular therapies (e.g., chimeric antigen receptor T-cell [CAR-T]-cell therapy and bispecific antibodies) (3–6). These advances have significantly improved survival outcomes. However, MM remains incurable, and managing patients with relapsed/refractory multiple myeloma (RRMM), particularly those refractory to multiple drug classes, continues to pose a major clinical challenge.

Carfilzomib, a selective and irreversible proteasome inhibitor, received US Food and Drug Administration (FDA) approval in 2012 and National Medical Products Administration (NMPA) approval in China in 2021 (7, 8). Pomalidomide, a potent third-generation IMiD, was FDA-approved in 2013 and NMPA-approved in 2020 for RRMM patients with ≥ 2 prior therapies, including lenalidomide and bortezomib (9, 10). The combination of carfilzomib and dexamethasone (Kd) forms a backbone regimen that is often combined with lenalidomide (KRd) or pomalidomide (KPd) to enhance efficacy (11–16).

In the current treatment era, dominated by CD38 monoclonal antibody-based frontline and early-relapse therapies and the increasing use of cellular therapies in later lines, identifying effective regimens for patients refractory to or relapsing after these novel agents is critical. KPd represents a valuable PI/IMiD combination option, particularly for lenalidomide-refractory patients. For example, among patients experiencing first progression during lenalidomide maintenance after autologous stem cell transplantation (ASCT), KPd achieved an overall response rate (ORR) of 92% and a median progression-free survival (PFS) of 26 months (13).

Despite robust evidence from clinical trials, real-world data on KPd efficacy and safety in contemporary practice, specifically for patients heavily pretreated with CD38 antibodies and/or cellular therapies, remain scarce (17). Patients encountered in routine practice often present with more complex disease biology, poorer performance status, and greater treatment refractoriness than trial participants, which may affect outcomes.

Therefore, evaluating the real-world effectiveness of KPd in Chinese RRMM patients—many of whom have progressed after prior CD38 antibody exposure and before or after cellular therapy—is essential. This study retrospectively assessed the efficacy and safety of KPd in RRMM patients treated at two Chinese medical centers and analyzed the clinical factors influencing treatment response.

## Methods

### Study design and patient selection

This retrospective observational study was conducted at two medical centers in China: Shangyu People’s Hospital of Shaoxing and Taizhou Hospital of Zhejiang Province. Consecutive RRMM patients who received at least three cycles of KPd combination therapy between October 2022 and December 2024 were included. Patients who received fewer than three cycles due to loss to follow-up were excluded to ensure evaluable response data. Eligible patients were at least 18 years of age, had a confirmed diagnosis of MM according to the International Myeloma Working Group (IMWG) criteria (18), and experienced disease progression or relapse following at least one prior line of therapy, as defined by the IMWG criteria. Refractory disease was defined as progression during therapy. The final follow-up occurred in April 2025. Relevant clinical and laboratory data, including demographics, baseline disease characteristics, detailed treatment history, therapeutic interventions, and clinical outcomes, were systematically extracted from the electronic medical records of the participating institutions.

### Treatment regimen and assessments

Patients received the KPd regimen in 28-day cycles. Carfilzomib was administered intravenously at 20 mg/m^2^ on days 1 and 2 of cycle 1, with the dose increased to 27 mg/m^2^ on days 8, 9, 15, and 16 of cycle 1 and in all subsequent cycles. Dexamethasone (20 mg) was administered orally or intravenously on days 1, 2, 8, 9, 15, 16, 22, and 23 of each cycle. Pomalidomide (4 mg) was administered orally once daily on days 1 through 21 of each cycle. Decisions regarding dose modifications, supportive care measures, and adverse event (AE) prophylaxis and management were made by the treating physician on the basis of individual patient tolerance and institutional protocols.

The primary study endpoints were safety, PFS, and ORR. PFS was defined as the duration from initiation of KPd treatment to the date of documented disease progression or relapse according to the IMWG criteria, death from any cause, or the last confirmed follow-up, whichever occurred earliest. ORR was defined as the proportion of patients who achieved a partial response (PR) or better (i.e., PR, very good partial response [VGPR], complete response [CR], or stringent complete response [sCR]) as the best response, based on the IMWG criteria. Treatment response was assessed at the end of each cycle or upon clinical suspicion of relapse. The secondary endpoint was overall survival (OS), measured from KPd initiation until death from any cause or the last follow-up. For safety, all AEs were recorded and graded according to the National Cancer Institute Common Terminology Criteria for Adverse Events, version 5.0 (CTCAE v5.0). Cytogenetic risk was assessed using fluorescence *in situ* hybridization (FISH); high-risk cytogenetics were defined as the presence of del(17p), t(4;14), t(14;16), t(14;20), or amplification of chromosome 1q (1q21).

### Statistical analysis

Differences in ORR between patient subgroups were analyzed using the Chi-square test and logistic regression, with results reported as odds ratios (ORs) accompanied by their corresponding 95% confidence intervals (CIs). Time-to-event endpoints, specifically PFS and OS, were estimated using the Kaplan–Meier method. A two-sided *p*-value less than 0.05 was considered statistically significant in all inferential tests. All statistical analyses were performed using R software, version 4.5.1.

## Results

### Patient characteristics

Thirty-four patients received KPd therapy within the study period (**Table 1**). The median age at multiple myeloma diagnosis was 65 years (range: 46–81), with men comprising 61.8% (*n* = 21) of the cohort. Over half (52.9%, *n* = 18) presented with International Staging System (ISS) stage III disease, and IgG myeloma was the predominant subtype (55.9%). Cytogenetic risk assessment was performed in 16 patients, with high-risk features observed in 29.4% (*n* = 10). Patients had received a median of two prior lines of therapy (range: 1–8). Previous treatments included ASCT in 35.3% (*n* = 12) of patients and B-cell maturation antigen (BCMA)-directed CAR-T therapy in 8.8% (*n* = 3). Among the heavily pretreated population, all patients had prior bortezomib exposure (100%), most had lenalidomide exposure (91.2%, *n* = 31), and half had daratumumab exposure (50.0%, *n* = 17). Notably, 15 patients received daratumumab as second-line treatment. Triple-class exposure was documented in 41.2% (*n* = 14).

**Table 1:** Patient characteristics.

Characteristics	Overall (*n* = 34)
Sex	
Female	13 (38.2%)
Male	21 (61.8%)
Age (years)	
Median (range)	65 (46–81)
≥ 65	18 (52.9%)
< 65	16 (47.1%)
Disease stage ISS	
Stage I	5 (14.7%)
Stage II	11 (32.4%)
Stage III	18 (52.9%)
Subtype	
IgG	19 (55.9%)
IgA	8 (23.5%)
Light chain	4 (11.8%)
Others^a^	3 (8.8%)
Cytogenetics	
Standard risk	6 (17.6%)
High risk^b^	10 (29.4%)
Not available	18 (52.9%)
Previous treatment lines	
1	15 (44.1%)
2	14 (41.2%)
≥ 3	5 (14.7%)
Median	2
Range	1–8
Previous treatment type	
Bortezomib	34 (100.0%)
Lenalidomide	31 (91.2%)
Daratumumab	17 (50.0%)
CAR-T cell	3 (8.8%)
Previous ASCT	
No	22 (64.7%)
Yes	12 (35.3%)
Lactate dehydrogenase	
≤ ULN	27 (79.4%)
> ULN	7 (20.6%)
eGFR	
< 60 mL/min/1.73 m^2^	21 (61.8%)
≥ 60 mL/min/1.73 m^2^	13 (38.2%)

*ISS*, International Staging System; *CAR*, chimeric antigen receptor; *ASCT*, autologous stem cell transplantation; *ULN*, upper limit of normal; *eGFR*, estimated glomerular filtration rate.

^a^ Include IgD (*n* = 2) and biclonal subtype (*n* = 1).

^b^ del(17p), t(14;16), t(4;14), t(14;20), or amplification of chromosome 1q.

### Treatment efficacy

KPd therapy induced a partial response or better (ORR) in 28 patients (82.4%). The depth of response included a CR or better in six patients (17.6%) and a VGPR in 13 patients (38.2%). Significant associations were observed between a lower ORR and two clinical factors: prior daratumumab exposure (OR: 0.05; 95% CI: 0.00–0.99) and receipt of ≥ 3 prior lines of therapy (OR: 0.10, 95% CI: 0.01–1.05) (**Table 2**). The ORR did not significantly differ across patient subgroups according to sex, age (≥ 65 years), ISS stage, cytogenetics data, baseline lactate dehydrogenase, estimated glomerular filtration rate (eGFR), or prior exposure to lenalidomide, CAR-T-cell therapy, or ASCT.

**Table 2:** Association of patient characteristics with the overall response rate.

Subgroup	*n* (event *n*)	Response rate (%)	ORR in univariate analysis	
			OR (95% CI)	*p*-value
Sex				
Female	13 (10)	76.9	1.00 (reference)	0.849
Male	21 (18)	85.7	1.80 (0.30–10.64)	
Age (years)				
≥ 65	18 (15)	83.3	1.00 (reference)	1
< 65	16 (13)	81.2	0.87 (0.15–5.06)	
Disease stage ISS				
Stage I	5 (5)	100.0	1.00 (reference)	0.235
Stage II	11 (10)	90.9	0.64 (0.02–18.37)	
Stage III	18 (13)	72.2	0.22 (0.01–4.76)	
Cytogenetics				
Standard risk	6 (6)	100.0	1.00 (reference)	0.072
High risk^a^	10 (6)	60.0	0.11 (0.00–2.51)	
Not available	18 (16)	88.9	0.51 (0.02–12.08)	
Previous treatment lines				
1	15 (13)	86.7	1.00 (reference)	0.024^*^
2	14 (13)	92.9	2.00 (0.16–24.87)	
≥ 3	5 (2)	40.0	0.10 (0.01–1.05)	
Previous lenalidomide				
No	3 (2)	66.7	1.00 (reference)	
Yes	31 (26)	83.9	2.60 (0.20–34.46)	
Previous daratumumab				
No	17 (17)	100.0	1.00 (reference)	0.024^*^
Yes	17 (11)	64.7	0.05 (0.00–0.99)	
Previous CAR-T				
No	31 (26)	83.9	1.00 (reference)	1
Yes	3 (2)	66.7	0.38 (0.03–5.10)	
Previous ASCT				
No	22 (17)	77.3	1.00 (reference)	0.561
Yes	12 (11)	91.7	3.24 (0.33–31.54)	
Lactate dehydrogenase				
≤ ULN	27 (21)	77.8	1.00 (reference)	0.413
> ULN	7 (7)	100.0	4.53 (0.23–90.56)	
eGFR				
< 60 mL/min/1.73 m^2^	21 (18)	85.7	1.00 (reference)	0.849
≥ 60 mL/min/1.73 m^2^	13 (10)	76.9	0.56 (0.09–3.29)	

*ORR*, overall response rate; *OR*, odds ratio; *ISS*, International Staging System; *CAR*, chimeric antigen receptor; *ASCT*, autologous stem cell transplantation; *ULN*, upper limit of normal; *eGFR*, estimated glomerular filtration rate.

^*^ Statistically significant (*p* < 0.05).

^a^ del(17p), t(14;16), t(4;14), t(14;20), or amplification of chromosome 1q.

### Survival outcomes

**Figure 1:**
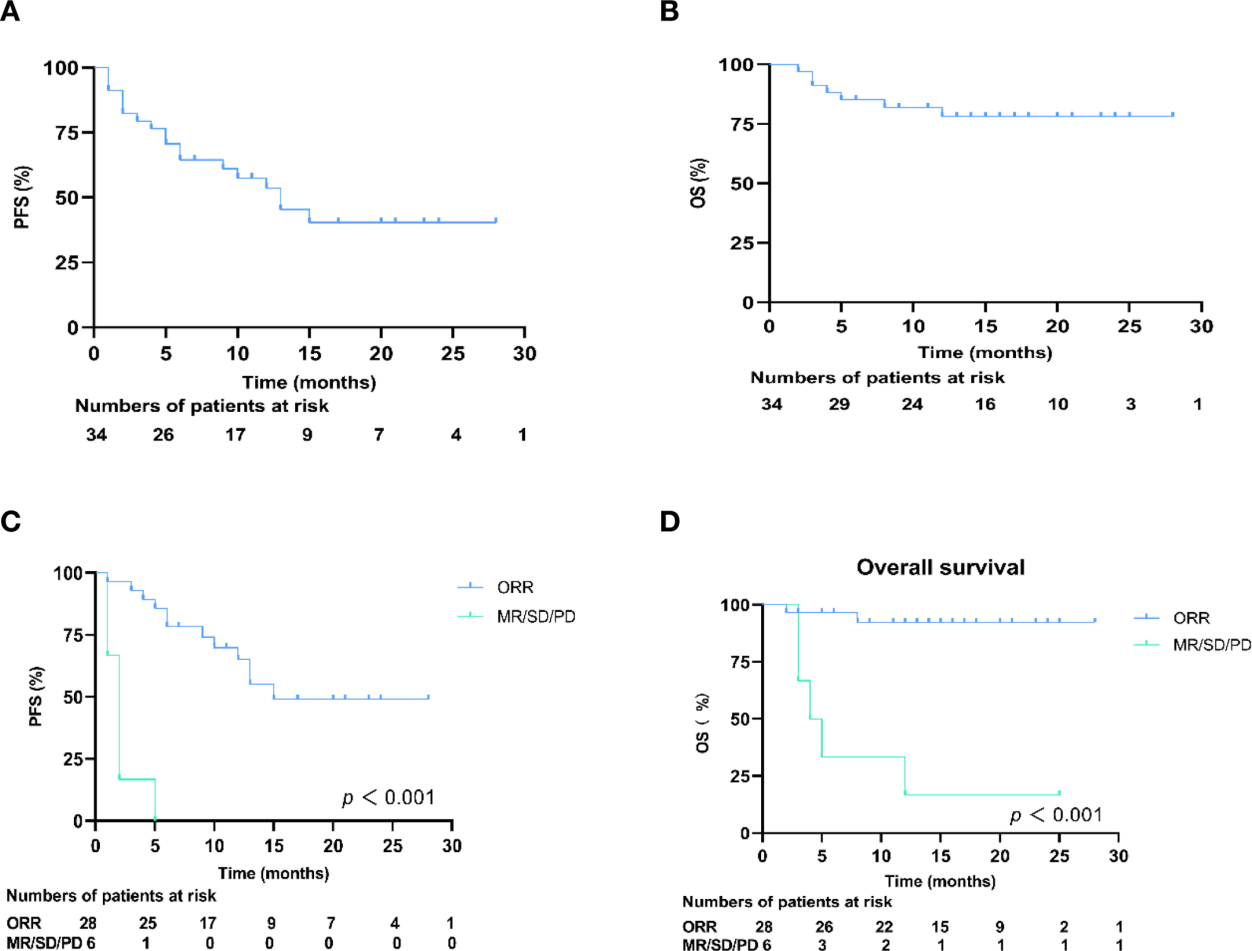
PFS and OS for patients who received KPd therapy. **(A)** PFS for patients who received KPd therapy. **(B)** OS for patients who received KPd therapy. **(C)** PFS for nonresponders vs. patients achieving an objective response. **(D)** OS for nonresponders vs. patients achieving an objective response.

At a median follow-up of 16 months (range: 2–28), the median PFS was 13 months. The PFS rate was 53.6% at 1 year and 40.3% at 2 years (**Figure 1a**). The median OS was not reached; the 1-year OS rate was 78.1% (**Figure 1b**). In total, 20.6% (*n* = 7) of patients died, primarily due to progressive disease (14.7%, *n* = 5) or infection (5.9%, *n* = 2). Compared with nonresponders, patients with an objective response (≥ PR) exhibited significantly superior PFS and OS (*p* < 0.001) (**Figures 1c, d**). A trend toward reduced survival was noted in daratumumab-exposed patients.

### Safety profile

Treatment-related AEs affected 88.2% (*n* = 30) of patients, with grade 3/4 events occurring in 58.8% (*n* = 20). The predominant hematologic toxicities were as follows: neutropenia (73.5% overall, 52.9% grade 3/4), thrombocytopenia (52.9% overall, 23.5% grade 3/4), and anemia (29.4% overall, 11.7% grade 3/4) (**Table 3**). Infections were documented in 35.3% (*n* = 12), including four grade 3/4 events and two fatal outcomes. Renal injury occurred in 35.3% (*n* = 12), including two cases of grade 4 acute renal failure. Other notable AEs included gastrointestinal events (32.4%, *n* = 11), metabolic dysfunction (23.5%, *n* = 8), hepatotoxicity (20.6%, *n* = 7), cardiovascular events (14.7%, *n* = 5), and peripheral neuropathy (5.9%, *n* = 2).

**Table 3:** Adverse events (AEs) during treatment.

Adverse events	Any grade	Grade ≥ 3
Hematological AEs		
Neutropenia	25 (73.5%)	18 (52.9%)
Thrombocytopenia	18 (52.9%)	8 (23.5%)
Anemia	10 (29.4%)	4 (11.7%)
Nonhematological AEs		
Infections	12 (35.3%)	4 (11.7%)
Renal injury	12 (35.3%)	2 (5.9%)
Gastrointestinal events	11 (32.4%)	0
Metabolic dysfunction	8 (23.5%)	2 (5.9%)
Hepatotoxicity	7 (20.6%)	1 (2.9%)
Cardiovascular events	5 (14.7%)	0
Peripheral neuropathy	2 (5.9%)	0

## Discussion

This real-world analysis demonstrated the clinical utility of KPd in Chinese patients with RRMM, a population historically underrepresented in registrational trials. The observed ORR of 82.4% and ≥ VGPR rate of 55.9% align with the established efficacy profile of KPd but show some key distinctions from pivotal studies. The phase II trial supporting the approval of KPd reported higher response rates (ORR: 92%, ≥ VGPR: 75%) (13), which may be attributable to fundamental differences in patient characteristics. Our cohort exclusively comprised heavily pretreated individuals (median: 2 prior lines; range: 1–8), including 50% daratumumab-exposed patients, 29.4% with high-risk cytogenetics, and transplant-ineligible patients—populations systematically excluded from earlier trials. The significant negative effects of prior daratumumab exposure (OR: 0.05) and ≥ 3 treatment lines (OR: 0.10) on the ORR underscore how real-world efficacy is affected by disease complexity and prior therapeutic pressure.

Survival outcomes further validated the clinical value of the KPd regimen in this setting. The median PFS of 13 months and 2-year PFS rate of 40.3% compare favorably with historical data for similarly pretreated RRMM populations (19), particularly given that 91.2% of patients were lenalidomide-refractory and 50% were daratumumab-exposed. Of the three patients who received prior CAR-T therapy, none progressed within 3 months post-CAR-T; the time from CAR-T to disease progression was 5, 20, and 27 months, respectively. The patient who progressed at 5 months did not respond to KPd, whereas the other two achieved a response.

The mechanisms underlying these effects in post-CAR-T settings warrant further investigation, but the results suggest potential synergy between proteasome inhibition and immune reconstitution after T-cell therapy.

The observed safety profile reinforces known KPd toxicity while highlighting context-specific challenges. Hematologic AEs dominated our analysis (73.5% neutropenia, 52.9% thrombocytopenia), exceeding rates reported in clinical trials (20, 21), a finding attributable to cumulative marrow suppression in heavily pretreated patients and reduced dose-intensity tolerance. The incidence of nonhematologic toxicities aligned with expectations, although the 35.3% incidence rate of renal injury indicates a need for increased vigilance, especially given MM’s propensity for renal impairment. Proactive management (e.g., hydration protocols and dose adjustments for eGFR < 30 mL/min) is essential. Conversely, the lower cardiovascular AE rate (14.7% vs. the literature (22–24)) likely reflects stringent cardiac screening preenrollment—a practice that merits standardization.

The results should be carefully interpreted with consideration of the study limitations: The retrospective design introduces potential selection bias, while the modest sample size (*n* = 34) limits multivariate analysis of prognostic factors. Moreover, the requirement of at least three cycles could have excluded those with early toxicities, potentially underestimating the incidence of grade 3–4 adverse events. The availability of cytogenetic data for only 47% of patients restricts risk-stratified conclusions. Heterogeneity in postresponse maintenance therapies may have confounded survival outcomes, and the median 16-month follow-up precluded mature overall survival analysis. Future prospective studies should address these constraints while exploring biomarkers (e.g., 1q21 amplification impact) and sequencing strategies relative to novel agents.

## Conclusion

KPd appears to show promising activity in RRMM in real-world practice, showing potential efficacy in Chinese patients refractory to lenalidomide, proteasome inhibitors, anti-CD38 antibodies, and CAR-T-cell therapy. While hematologic toxicity must be vigilantly monitored and preemptively managed, the manageable safety profile of KPd supports its inclusion in contemporary therapeutic sequences. As novel modalities reshape RRMM treatment paradigms, KPd provides a critical bridge for patients with multirefractory disease, particularly where access to advanced cellular therapies remains limited.

## Data Availability

The raw data supporting the conclusions of this article will be made available by the authors, without undue reservation.
